# Medical News and Items

**Published:** 1877-11

**Authors:** 


### Medical News and Items.

Karl August Wunderlich, the well known professor and clinical teacher at Leipzig, Germany, is dead.

Married—At West Brooklyn, Ill., by the Rev. C. H. Hoffman, Gustavus F. Schreiber, M. D., and Miss Lettie Morey, all of West Brooklyn.

The Library of the Medical Press Association is under great obligations to Dr. Rauch, President of the State Board of Health, for the donation of the Transactions of the American Public Health Association.

Central Free Dispensary of West Chicago, corner Wood and Harrison Streets.—Statement of dispensary work for September: Vaccinations performed, 454; total number of patients, 1,329; number of visits of patients to the dispensary, 2,259; number of visits to the homes of patients, 343; total number of visits, 2,602; number of prescriptions furnished, 2,377.

Mortality in Chicago during the Four Weeks Ending October 15.—Total number of deaths, 564; males, 312; females, 252; under one year, 142. Principal causes of death: Accidents, 19; apoplexy, 4; cholera infantum, 29; diarrhœa, 32; dysentery, 11; enteritis, 7; convulsions, 57; scarlatina, 37; variola, 5; diphtheria, 23; typhoid fever, 30; puerperal fever 2; phthisis, 56; pneumonia, 15; meningitis, 16; diseases of heart, 13; liver, 2; kidneys, 6.

We have received a printed excerpt from the *Clinton County* (Iowa) *Advertiser*, of Oct. 11, 1877, giving the details respecting the arrest and punishment of a swindler styling himself Dr. A. G. Walter (alias Williard, alias Bayreuz). He succeeded in fraudulently procuring thirty-one dollars and sixty-five cents from Dr. Chas. H. Lathrop, of Lyons, Iowa, on the ground that he had been commissioned by the "United States College of Surgeons, Washington, D. C.,"—an institution endowed by the general government for the purpose of relieving those afflicted with paralysis and epilepsy. Dr. Lathrop was suffering from paraplegia, and paid the sum named for the purpose of being enrolled as a patient in the institution which it was pretended had been established.

We fully sympathize with Dr. Lathrop, but must express the difficulty we find in understanding how a U. S. Examining Surgeon for Pensions, could be entrapped by such a flimsy and transparent device.

MILITARY SURGERY IN THE TURKISH ARMY.

An artillery-man had a limb shattered by an exploding shell at Sistova, and, incredible as it may appear to those unfamiliar with the organization of the Turkish administration, the unfortunate man had to be transported from Sistova to Constantinople. In spite of his terrible suffering he listened to the latest war news with the keenest interest. Amputation having been decided upon, authority to perform the operation was requested of the minister of war, for, in accordance with the curious customs which the student of the administrative régime of that country is always discovering, no amputation can be performed in a Turkish hospital without official permission. It, of course, frequently happens that the patient dies, while incompetent authorities are deliberating upon the formal demand of the surgeon. Our artillery-man was fortunate in having the consideration of the question respecting his leg conducted with exceptional rapidity, as a conclusion was reached in eight or ten days! The brave fellow who had waited to learn the good pleasure of the administration all the way from Sistova, did not complain of this delay in the least. He finally endured the amputation with heroic courage, and it is hoped he may be saved.—(Le Temps.)

The *Hospital Gazette* and the *Archives of Clinical Surgery* have been consolidated, and form a very creditable semi-monthly journal of twenty-four pages. A further reduction of the number of medical periodicals in this country would greatly enhance the character and value of our periodical literature.

The list of licentiated physicians we publish in this issue, comprises practitioners in Chicago and Cook county. Only about 300 physicians in this county have, so far, complied with the provisions of the law.

In our next number we shall give the official list of licentiates from other counties.

On and after November 1st, the State Board of Health will be in session at the Grand Pacific Hotel, Chicago, to examine candidates for licence. And on the 15th of November, the board will hold a meeting in Cairo, for the same purpose.

Under the head of "Honors to an American," the *St. Louis Clinical Record* makes the following very severe statements, which, if true, ought to be very generally known, and if not true, ought to subject the editor of the *Record* to damages for libel:—

"Several of our contemporaries are giving great prominence to Dr. Sayre's very flattering reception in England. It seems that Dr. Sayre went to England to advertise his (*sic.*) method of treating spinal curvature. He intends to publish a book describing his (?) process and expects a large sale under an English copyright.

"This would be all very well—in fact, just as it should be—if Dr. Sayre had ever invented anything, which he never did, so far as we are informed.

"'Dr. Sayre's hip-joint splint' was invented by Dr. Davis.

"'Dr. Sayre's plaster-of-Paris jacket' was invented and first applied by Dr. Bryan, of Lexington, Ky.

"'Dr. Sayre's method of self-suspension in rotary-lateral spinal curvature' was invented by Dr. Benj. Lee, of Philadelphia.

"'Dr. Sayre's lectures on orthopædic surgery' were by Dr. Louis Bauer, formerly of Brooklyn, N. Y., now of St. Louis.

"As a plagiarist and 'father of other men's ideas,' Dr. Sayre is without a rival. We are glad to see that our English cousins delight to honor such representative Americans (Heaven save the mark!) as P. T. Barnum and L. A. Sayre. *Vive le humbug!*"—(*Phil. Med. Times.*)

Opening of the Colleges.—The regular session of the *Chicago Medical College* was opened with the usual public exercises, on the evening of the 1st inst. Prof. J. S. Jewell delivered the opening address. His effort was a variation upon the hackneyed subject for college openings of "how to to succeed in the chosen profession." He spoke of "the course men usually take to fail in the profession," etc.

The size of the class of this college is larger than that of last year, at the beginning of the session, notwithstanding the increase in the severity of the conditions of matriculation and graduation, voted last spring.

*Rush College* commenced her course of lectures with public exercises, on the evening of the 2d ult. Prayer was offered by Rev. Prof. Hyde, and President Allen spoke a few words on the lives, character and services of the three distinguished men who had preceded him in the presidential chair, Drs. Brainard, Blaney and Freer.

The regular address of the occasion was delivered by Prof. J. H. Etheridge, who had chosen for his subject, "Waste and Repair."

The class at the opening of the session is over 350.

*The Woman's Hospital Medical College* began her annual term with a jubilee and much felicitation on the evening of the 9th ult. Notwithstanding a drenching rain, the evening was a gala time for this institution. The college has a right to congratulate herself, and receive the congratulations of her friends and neighbors, for she has moved into a new house, which is her own, where she intends to stay. The college building on Lincoln street, opposite the County Hospital, which has been rebuilt for this institution, is the most complete and convenient medical college building we know of. It is large enough to accommodate a class of one hundred; has two lecture rooms, a chemical laboratory, dissecting room, microscope room, museum, all the necessary closets and ante-rooms, and a parlor, and a statue of *Minerva* crowning the building.

The Chairman of the Building Committee, Prof. T. D. Fitch, made a statement of the history of the organization of the faculty and the building of the present structure.

Prof. C. W. Earle gave the address of the evening, which dealt with the history of the college, and the subject of the education of women in medicine in Chicago.

Prof. W. E. Quine made a brief speech in behalf of the new members of the faculty; and Dr. Ephraim Ingals, in a a few happy impromptu words, congratulated the college, the faculty, and the movement for the medical education of women, on the fortunate auspices under which the college this year was beginning its lecture term.

About thirty students are matriculated at this institution.

THE MEDICO-LEGAL INVESTIGATION OF SPERMATOOZA.—Ferrand. (*La France Médicale*, Oct. 3, 1877). One of the most difficult tasks of legal medicine, is the characterization of seminal stains. When the stains are old and have been subjected to either friction or freezing, it is often well-nigh impossible to separate the spermatozooids, and to secure them upon the stage of the microscope in sufficiently good condition and number to justify a positive and final conclusion as to their character. Almost invariably the animalcules have been broken. The large extremity generally called "the head," is detached from the filiform appendix which is called "the tail," the various parts are more or less deformed; and the dimensions and general aspect of the whole equally suggest either debris of tissue or organic infusoriæ.

Longuet, after giving especial attention to this question, has recently communicated to the Society of Legal Medicine, the result of his researches. The procedures employed by him for the recognition of the spermatozooids, are so simple and practical, that they deserve to be given a wider publicity.

The first requisite on the part of the expert is a manipulation, which is the same, whatever method be finally adopted for the isolation of the elements. It consists in putting a piece of the solid material in contact with distilled water, so that by imbibition, the tissue itself and the material with which it is impregnated may become swollen. This done, it is placed on a slide of clean dry glass by the aid of needles, when it is carefully picked apart in such a manner as not to break any elements that may be present. Thread by thread, and fibril by fibril, the careful dissection is made until each form is isolated and subjected to a minute examination in turn under the objectives of the microscope. In order to render the whole more visible and to better limit the contour, a weak solution of iodine is used for the purpose of staining the tissue.

Too much precaution cannot be taken. The trouble with the procedure is, that however careful the expert may be, he is liable to create artificial spermatozooids which may be confounded with the real. "Certain vegetable fibrillæ, particularly those of hemp, contain in their interior, certain ovoid granulations, slightly flattened in their longest diameter and very refractive, which precisely resemble, in dimensions, aspect and form, the so-called head of the anamalcule. These granules become free as soon as the cellules are destroyed, and are dispersed in the liquid where the debris of the material is floating."

Longuet accordingly has searched for a coloring matter which by its elective action would permit one to distinguish between the anamalculæ and the vegetable detritus; and, after numerous essays, be succeeded with ammoniacal carmine, such as the histologists use. The spermatozooids behave differently in the presence of the carmine, according as they are fresh, or old and dry. When recent they are very slightly changed; when old, they fix the color with great intensity, more particularly in the large extremity, the so-called "tail" remaining uncolored. This singular property suffices for their immediate recognition, even when they are surrounded by foreign elements which affect analogous forms.

The author advises as follows:

1. Take a small square of the material, supposed to be stained with semen, cut as nearly as possible from the centre of the stain.

2. Plunge the little square of material into a small quantity of distilled water, colored by a few drops of an ammoniacal solution of carmine (5 or 6 drops to 5 grammes of water).

3. Leave this to macerate for 36 or 48 hours, and even more, for no inconvenience will result.

4. Separate the threads of the material very carefully, thread by thread and fibre by fibre.

5. Isolate each separate fibre.

6. Examine each separately by the microscope, with an enlargement of 500 diameters, each morsel being placed in a drop of ordinary glycerine.

In a preparation thus made, clusters of spermatozooids will be seen for the most part entire, "the head" colored a light red, "the tail," without tint, disseminated among non-coloured vegetable fibrillæ of perfect refraction.

The advantage of this method lies in the fact that the results are most decisive according as the stain is old—that is, under the most unfavorable circumstances, for nothing is more easy than the isolation and recognition of spermatozooids in recent stains.

